# Pressure accelerates the circadian clock of cyanobacteria

**DOI:** 10.1038/s41598-019-48693-1

**Published:** 2019-08-27

**Authors:** Ryo Kitahara, Katsuaki Oyama, Takahiro Kawamura, Keita Mitsuhashi, Soichiro Kitazawa, Kazuhiro Yasunaga, Natsuno Sagara, Megumi Fujimoto, Kazuki Terauchi

**Affiliations:** 1 College of Pharmaceutical Sciences, Ritsumeikan University, 1-1-1 Nojihigashi, Kusatsu, Shiga 525-8577 Japan; 2 Graduate School of Life Sciences, Ritsumeikan University, 1-1-1 Nojihigashi, Kusatsu, Shiga 525-8577 Japan; 3 College of Life Sciences, Ritsumeikan University, 1-1-1 Nojihigashi, Kusatsu, Shiga 525-8577 Japan

**Keywords:** Molecular conformation, Supramolecular assembly, Thermodynamics

## Abstract

Although organisms are exposed to various pressure and temperature conditions, information remains limited on how pressure affects biological rhythms. This study investigated how hydrostatic pressure affects the circadian clock (KaiA, KaiB, and KaiC) of cyanobacteria. While the circadian rhythm is inherently robust to temperature change, KaiC phosphorylation cycles that were accelerated from 22 h at 1 bar to 14 h at 200 bars caused the circadian-period length to decline. This decline was caused by the pressure-induced enhancement of KaiC ATPase activity and allosteric effects. Because ATPase activity was elevated in the CI and CII domains of KaiC, while ATP hydrolysis had negative activation volumes (Δ*V*^≠^), both domains played key roles in determining the period length of the KaiC phosphorylation cycle. The thermodynamic contraction of the structure of the active site during the transition state might have positioned catalytic residues and lytic water molecules favourably to facilitate ATP hydrolysis. Internal cavities might represent sources of compaction and structural rearrangement in the active site. Overall, the data indicate that pressure differences could alter the circadian rhythms of diverse organisms with evolved thermotolerance, as long as enzymatic reactions defining period length have a specific activation volume.

## Introduction

Circadian rhythms are endogenous timing systems that induce the circadian clock, resulting in numerous organisms, from cyanobacteria to higher animals, being adapted to the day-night cycle^1,2^. The circadian clock of cyanobacteria is the only model clock that has been reconstituted *in vitro*. It is consisted of KaiA, KaiB, and KaiC proteins and adenosine triphosphate (ATP)^3–6^. Homologues of the *kai* genes are widely distributed in various photosynthetic and non-photosynthetic bacteria^2^. KaiC forms a homo-hexamer that forms the two stacked rings of domains CI and CII. KaiC is able to autophosphorylate and autodephosphorylate S431 and T432. It also exhibits self-sustainable oscillation of phosphorylation *in vitro* when incubated with KaiA, KaiB, and ATP. KaiA enhances KaiC autophosphorylation, while KaiB attenuates the effect of KaiA^7–10^. Therefore, the KaiC phosphorylation cycle depends on these dynamics for assembly, in association with KaiC autophosphorylation and autodephosphorylation^11,12^. Furthermore, the *in vitro* circadian phosphorylation rhythm of KaiC compensates for temperature changes, is entrained via the temperature cycle^4^, and is strongly correlated with KaiC ATPase activity associated with KaiC kinase activity^13^. The ATPase activity of KaiC is 10^3^ to 10^7^ times lower than that of other well-known motor proteins^14^. Although ATPase activity in the CII domain is directly associated with kinase activity in the domain, ATPase activity in the CI domain dominates over CII-ATPase activity; consequently, CI-ATPase is considered the most fundamental parameter in defining the oscillation period *in vitro*^14,15^. However, our understanding remains limited about why KaiC-ATPase has such low reactivity and allosteric regulation, i.e. the interaction between CI-ATPase and kinase activities.

In addition to temperature, pressure is an important physical factor regulating protein structure, stability, and activity; however, how hydrostatic pressure affects biological rhythms, including the circadian rhythms of organisms, remains unknown. Pressure-axis experiments are advantageous because they facilitate investigations on deep-sea organisms and their proteins. More generally, unlike temperature and denaturing agents, the effects of pressure on protein structures and chemical reactions in solution depend solely on a volume change in the system^16–18^. For instance, open conformations of enzymes, wherein internal cavities are exposed and hydrated, might be stabilized under high pressure, because the partial molar volume of the open and hydrated protein is generally smaller than that of the well-folded, closed conformation^19,20^. In addition, pressure affects the rate of reactions. A reaction requiring a positive activation volume is decelerated under high pressure. Conversely, a reaction requiring a negative activation volume is accelerated under high pressure^20^. Therefore, investigations on high-pressure reaction kinetics help theorize expansions and contractions in molecular structures that are in the transition state. Because such volume fluctuations in proteins are correlated with allosteric effects in proteins^21–23^, a pressure approach could be used to assess proteins undergoing allosteric regulation. The present study aimed to investigate the responses of the circadian phosphorylation cycle of KaiC from the cyanobacterium *Synechococcus elongatus* PCC 7942 and associated ATPase activity to hydrostatic pressure. Based on the volume fluctuation of KaiC, we expect to provide insights on the mechanism underlying the pressure-induced acceleration of the phosphorylation cycle and the allosteric regulation of KaiC in cyanobacteria.

## Results

### Pressure accelerates the circadian clock of cyanobacteria

**Figure 1:**
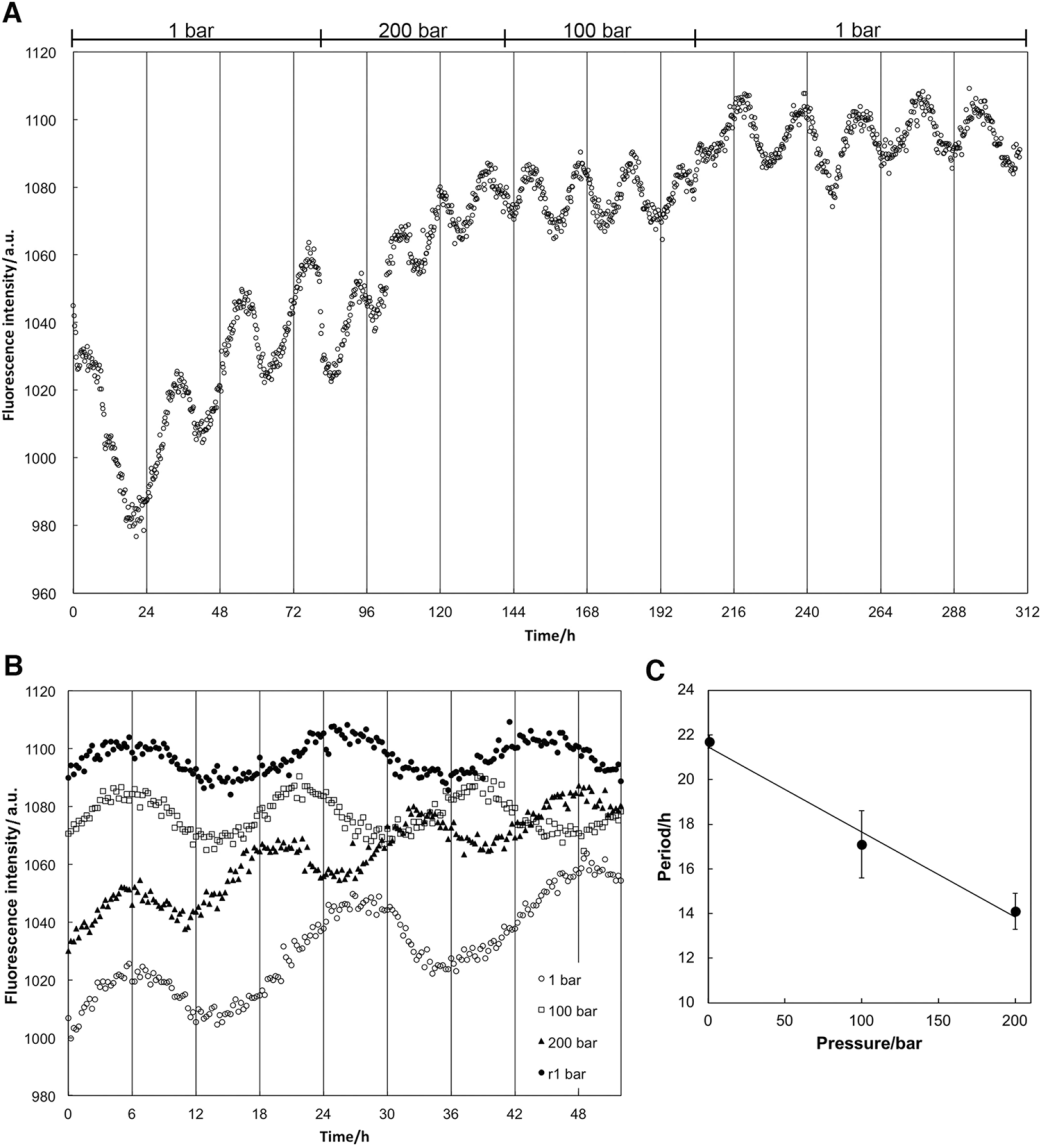
Pressure-induced changes to Trp fluorescence intensity during the KaiC phosphorylation cycle at 30 °C. (**A**) Oscillation of fluorescence intensity at different pressures. (**B**) Comparison of oscillation of fluorescence intensity at 1 bar, 100 bars, 200 bars, and 1 bar after pressure was released. (**C**) Pressure dependence of the period length of the fluorescence oscillation cycle.

KaiC was co-incubated with KaiA and KaiB in 20 mM Tris-HCl buffer (pH 8.0) containing 150 mM NaCl, 6 mM ATP, and 5 mM MgCl_2_. At 30 °C, we measured tryptophan (Trp) fluorescence of the solution every 15 min at 1 bar, 100 bars, and 200 bars (see Fig. 1A for example). On excitation at 295 nm, maximum Trp fluorescence intensity was 340 nm, and showed temporal oscillatory changes. The period length of Trp fluorescence measurements coincided with that of the KaiC phosphorylation cycle^24^. Because KaiA and KaiC have one and three Trp residues in their monomers, respectively, changes to fluorescence intensity are expected to reflect changes in protein conformation, inter-molecular interactions among the proteins, and the characteristic reactions of KaiC, including ATP hydrolysis, autophosphorylation, and autodephosphorylation. The mean oscillation period in several Trp fluorescence experiments was 21.7 ± 0.3 h (mean ± standard deviation) at 1 bar (n = 3), which was consistent with the KaiC phosphorylation cycle. Figure 1B shows the oscillation frequency of fluorescence intensity at 1 bar, 100 bars, 200 bars, and 1 bar after pressure was released. Unexpectedly, the oscillation frequency increased with increasing pressure. The mean period lengths at 100 bars, 200 bars, and 1 bar after decreasing pressure were 17.1 ± 1.5 (n = 2), 14.1 ± 0.8 (n = 2), and 21.0 ± 1.6 h (n = 3), respectively. Pressure-induced changes to period length were reversible within the experimental error margin. Assuming a monotonous reduction with an increase in pressure, the period length decreased by a factor of 3.8 h/100 bars (Fig. 1C).

**Figure 2:**
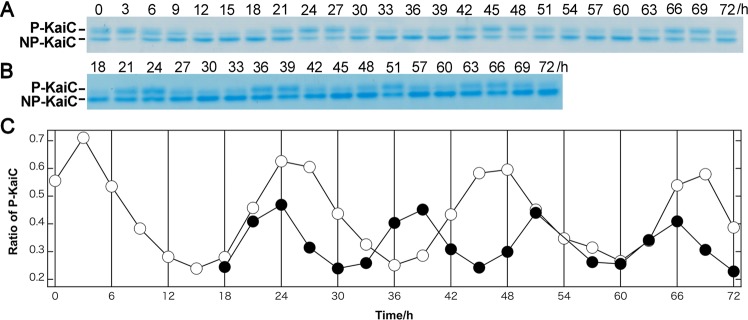
Oscillation of the phosphorylated KaiC ratio at 1 bar and 200 bars. (**A**,**B**) SDS-PAGE analyses of KaiC incubated at 1 bar (panel A) and 200 bars (panel B), respectively, with KaiA, KaiB, and ATP in silicon tubes. Pressure was applied at 15 h, where the phosphorylation rhythm of KaiC was entrained. Each reaction mixture in the silicon tubes was collected every 3 h and subjected to SDS-PAGE. P-KaiC and NP-KaiC indicate phosphorylated and unphosphorylated KaiC bands, respectively. Oscillation of the phosphorylated KaiC ratio was examined in triplicate. All of the full length gels are listed in a Supplementary Dataset File. (**C**) Changes to the ratio of phosphorylated KaiC bands at 1 bar (open circles) and 200 bars (closed circles).

We also measured pressure-induced changes in the ratios of phosphorylated KaiC to total KaiC in triplicate (see Fig. 2 for examples). Solution samples were incubated at 1 bar and 200 bars in the pressure-resistant vessel at 30 °C, and were collected every 3 h. Temporal changes to KaiC phosphorylation ratios were analysed via sodium dodecyl sulphate-polyacrylamide gel electrophoresis (SDS-PAGE) and ImageJ (National Institutes of Health, Bethesda, MD, USA). The period lengths at 1 bar and 200 bars were 21.8 ± 0.4 h (n = 3) and 13.3 ± 0.9 h (n = 3), respectively, which was consistent with those observed in Trp fluorescence measurements.

### Structure and thermodynamic stability of KaiA, KaiB, and KaiC at high pressure

To elucidate how pressure affects the structure and thermodynamic stability of Kai proteins, we measured the Trp fluorescence spectra of the proteins at 30 °C and at different pressures (from 1 bar to 300 bars). KaiA contains one Trp residue (i.e. W10) and KaiC contains three Trp residues in the CI (i.e. W92) and CII (i.e. W331 and W462) domains. Thus, Trp florescence is a suitable probe to monitor protein structure and stability. When pressure was increased from 1 bar to 300 bars, the fluorescence emission of KaiC, in terms of λ_max_ and intensity, remained largely unchanged (Fig. S1A). Because the phosphorylation state of KaiC alters during the cycle, we also investigated how pressure affects the KaiC phospho-mimic variants KaiC-DE (S431D/T432E) and -AA (S431A/T432A). These variants completely mimic phosphorylated and dephosphorylated states, respectively. Similar to wild-type (WT) KaiC, the fluorescence emission of the variants also remained largely unchanged at 300 bars (Fig. S1B, C). For KaiA, pressure had no noticeable effect on fluorescence emission at 300 bars (Fig. S2A). Because KaiB does not have any Trp residues, Tyr fluorescence spectra were measured from 1 bar to 300 bars. KaiB contains four Tyr residues (i.e. Y7, Y12, Y39, and Y93). Fluorescence emission of KaiB remained largely unchanged by pressure (Fig. S2B). Thus, at 300 bars, KaiA, KaiB, and KaiC retained their folded conformations during the KaiC phosphorylation cycle.

### Effect of pressure on the ATPase activity of KaiC

ATPase activity defines the period length of KaiC phosphorylation^13^. Because CI-ATPase dominates over CII-ATPase, CI-ATPase might set the pace of the circadian rhythm^14^. To evaluate the pressure dependence of ATPase activity in each domain, we generated an N-terminal domain fragment (CI-model) and an E77Q/E78Q variant (CII-model) of KaiC. We examined the ATPase activity of the proteins and WT KaiC at 1 bar, 100 bars, and 200 bars. Changes to ATP and ADP concentrations were measured via high-performance liquid chromatography (HPLC; Fig. S3A). Figure. S3B shows the representative kinetics of ADP production by WT KaiC at different pressures. The ATPase activity of the proteins, *k*_cat_, was estimated based on the rate constant for ADP production. The *k*_cat_ values of the proteins from 1 bar to 200 bars are listed in Table 1. The ATPase activity of WT KaiC was approximately 1.6-fold greater at 200 bars compared to 1 bar, wherein period length decreased by approximately 1.5-fold.

**Table 1:** ATPase activity of wild-type KaiC and its variants at different pressures.

Pressure/bar	*k*_cat_ ± stdev^a^/day^−1^ (n)^b^				
	KaiC-WT	CI domain (CI-ATPase)	KaiC E77QE78Q (CII-ATPase)	KaiC R393C	KaiC F470Y
1	14 ± 2 (17)	11 ± 2 (13)	2.3 ± 0.6 (11)	20 ± 3 (5)	21 ± 3 (4)
100	18 ± 4 (7)	12.0 ± 0.8 (5)	4 ± 1 (6)	n.d.	n.d.
200	22 ± 3 (10)	13 ± 1 (7)	5 ± 2 (6)	23 ± 2 (5)	25 ± 2 (4)
Ratio (200 bar/1 bar)	1.6	1.2	2	1.1	1.2

^a^Standard deviations. ^b^Number of protein samples. Concentration of each protein sample was separately adjusted, irrespective of the sample lot.

**Figure 3:**
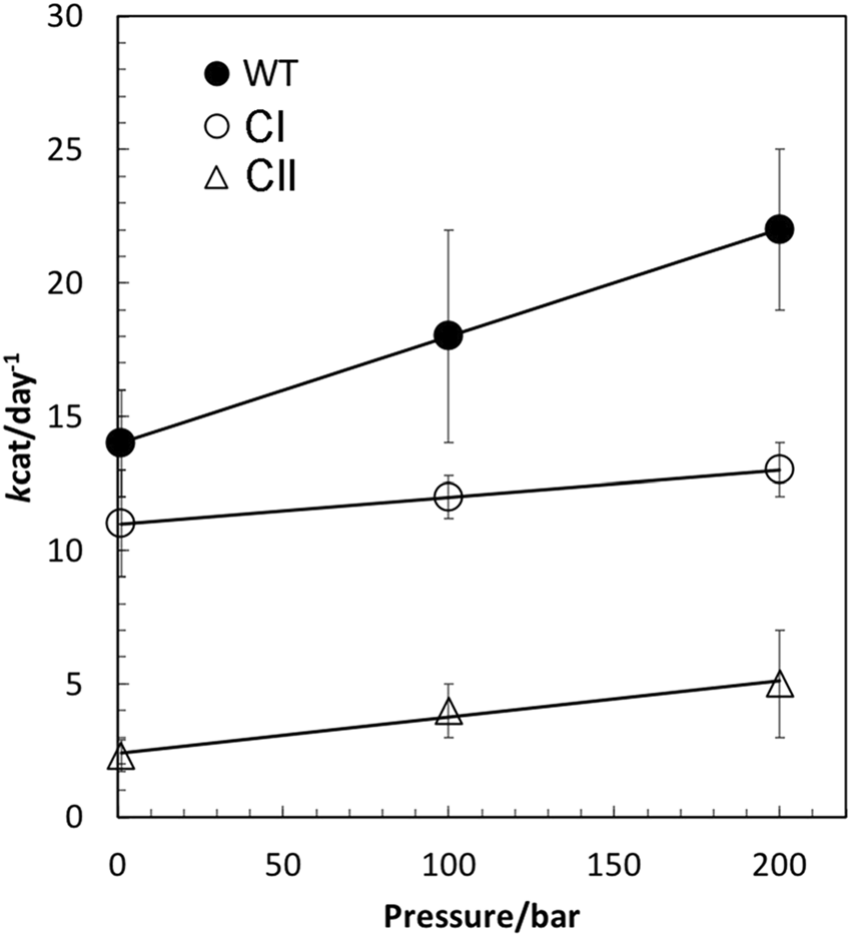
Pressure dependence of ATPase activity of KaiC. Pressure dependence of *k*_cat_ (day^−1^) for ATP hydrolysis of the wild-type (closed circles), the CI domain (CI-model) (open circles), and the E77Q/E78Q variant (CII-model) (open triangles) of KaiC at 30 °C. Data are presented in Table 1.

**Figure 4:**
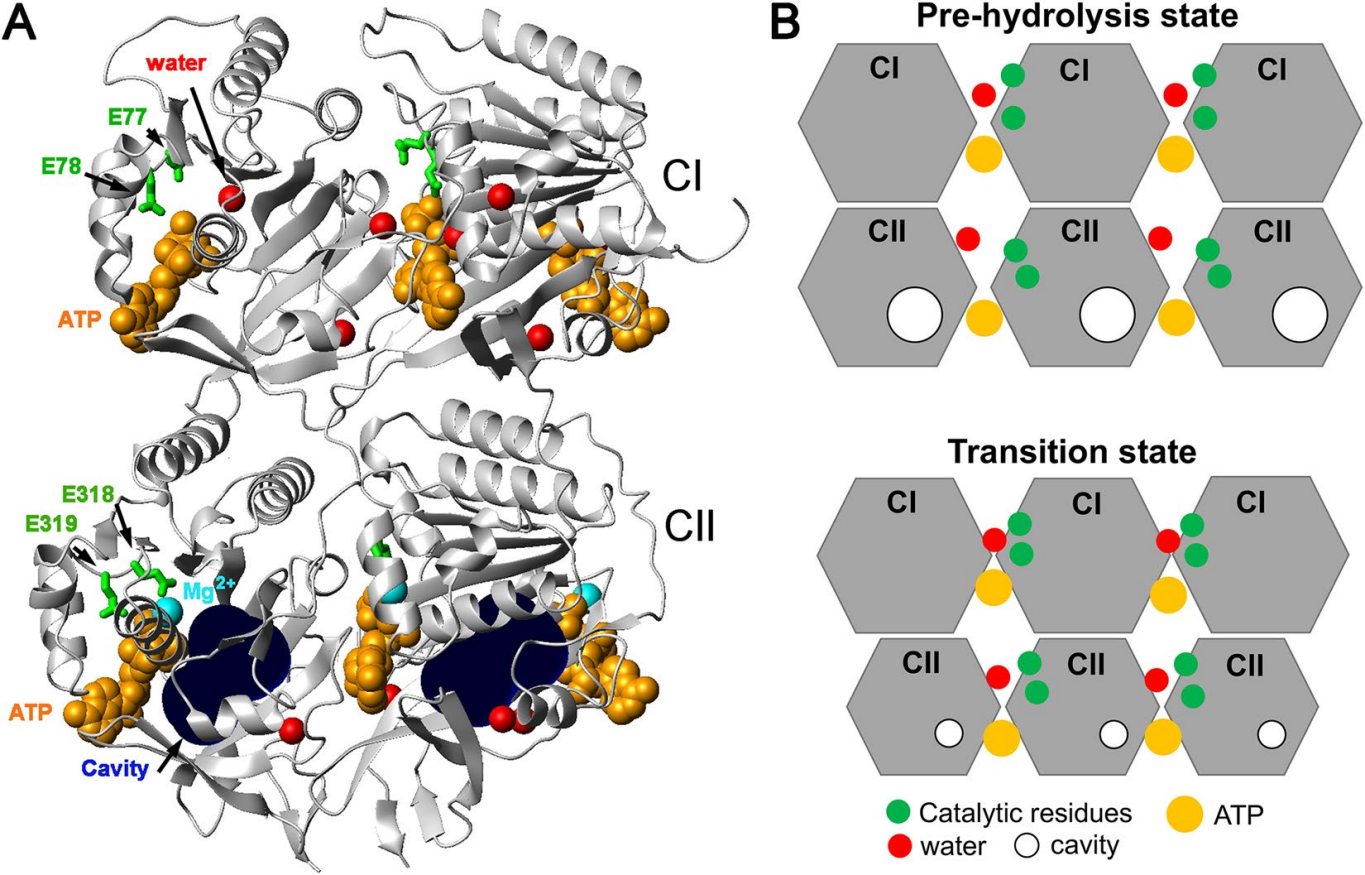
Internal cavities of KaiC and volume compaction on ATP hydrolysis. (**A**) Location of the cavity of the KaiC-ATP complex (PDB ID: 1U9I). Two subunits of KaiC (gray) and 6 ATP (gold) are depicted by ribbon and space-filling models, respectively. Mg^+2^ ions and water molecules are depicted by cyan and red spheres, respectively. Catalytic residues for ATP hydrolysis are depicted by green stick models (i.e., E77, E78, E318, and E319). Cavities are depicted by dark-blue spheres, and were estimated using the MOLMOL program^25^ with a 3.8-Å probe size. (**B**) Schematic of KaiC CI-CII domains of pre-hydrolysis and transition states. Three CI-CII domains are presented. Catalytic residues, ATP, water molecules, and cavities are depicted by green, yellow, red, and white circles, respectively.

Based on the pressure dependence of *k*_cat_ (Fig. 3), the activation volume required for ATP hydrolysis via the CI and CII domains was estimated to be −18 ± 2 mL/mol and −100 ± 30 mL/mol, respectively (Fig. S4). Evaluation of overall ATPase activity in KaiC showed that the activation volume was −57 ± 2 mL/mol. Interestingly, the CII-domain showed 5-fold higher volumetric contractions than the CI-domain on activation. To understand the origin of thermodynamic contraction, we investigated the internal cavities of the KaiC-ATP complex (PDB ID: 1U9I) using the MOLMOL program^25^. One large cavity was detected in each CII-domain of the KaiC-ATP complex when using a probe of 3.8-Å in size (Fig. 4A); however, small cavities were detected at all locations in KaiC when using a probe of 1.4 Å in size (Fig. S5).

## Discussion

This study demonstrated that hydrostatic pressure is positively correlated with a reduction in the length of the circadian period and the ATPase activity of KaiC. These results reflect previous reports on ATPase activity and period-length correlation among KaiC period-mutant proteins^13^. Therefore, pressure-induced acceleration of the phosphorylation cycle of KaiC might be attributed to the pressure-induced enhancement of its ATPase activity. Both the CI- and CII-models showed pressure-induced increments in ATPase activity, indicating that CII-ATPase contributes towards setting the pace of the circadian rhythm. The negative activation volume for ATP hydrolysis indicates that the transition-state (TS) has smaller partial molar volumes compared to the pre-hydrolysis state. Because the partial molar volume of globular proteins generally decreases with the compaction and/or hydration of internal cavities^17,19,26^, TS might have a more compact and/or hydrated conformation than the pre-hydrolysis state. Internal cavities, including the large cavity, serve as sources of compaction, hydration, and structural rearrangements in the active site of each CII-ATPase.

Akiyama *et al*. previously reported the osmolality dependence of the *in vitro* phosphorylation cycle of KaiC^27^. Specifically, with increasing glycerol concentrations of up to 4.5 mosm/kg H_2_O, corresponding to approximately 110 bar, period length was shortened to approximately 19 h^27^. The magnitude of osmotic pressure-induced changes in period length is consistent with that of the selected hydrostatic pressure in the present work. This co-occurrence indicates that the mechanism underlying the acceleration of the Kai oscillator is common under both types of pressure perturbation (i.e. hydrostatic and osmotic). Although the mechanism underlying this acceleration was previously unclear, the results of the current study clearly show that the pressure-induced acceleration of the circadian clock is attributed to an increase in KaiC ATPase activity.

Furthermore, Abe *et al*. reported that the retardation of ATP hydrolysis of KaiC is the result of the unfavourable position of a lytic water molecule at the active site, as well as the *cis*-to-*trans* isomerization of D145−S146 peptide coupled with ATP hydrolysis in the CI domain^14^. Based on previous studies and the results of the current study, we hypothesized that the thermodynamic contraction of the structure of the active site in TS favourably positions catalytic residues and lytic water molecules to facilitate ATP hydrolysis (Fig. 4B). According to this hypothesis, if catalytic residues and lytic water molecules are proximal to the active site (i.e. favouring ATP hydrolysis) in the pre-hydrolysis state, a less negative activation volume is required for the reaction. Indeed, CI-ATPase (which has higher activity than CII-ATPase) yielded a less negative activation volume than CII-ATPase. Of note, water molecules were detected at the active site of each CI domain in several crystals^14^; however, almost no water molecules were detected in the CII domains. Because a water molecule is essential for ATP hydrolysis, the difference in water distribution between the two domains might have been driven by protein dynamics and crystal packing. We applied this hypothesis to KaiC period variants, R393C (period length = 15 h) and F470Y (period length = 17 h)^13^, which contained a mutation in the CII domain. The ATPase activity of the variants at 1 and 200 bars is shown in Table 1. Assuming complete similarity in the CI-ATPase activity of the KaiC-variants with that of WT KaiC, the differences in ATPase activity among proteins could be attributed to their CII-ATPase activity. Because the CII-ATPase of the variants is expected to be more active than that of WT at 1 bar, less negative activation volumes for ATP hydrolysis are predicted in short-period mutants. Indeed, the relative ATPase activity of R393C and F470Y at a ratio of 200 bars to 1 bar (i.e. 200 bar/1 bar), whose natural logarithms are proportional to negative activation volumes, was calculated to be 1.1 and 1.2, respectively. These values were significantly less than that of WT (i.e. 1.6). These results also support our hypothesis.

Finally, allosteric effects on KaiC must be considered. A pressure approach is beneficial for proteins subject to allosteric regulation. The large volume contraction for CII-ATPase activity probably directly influences the kinase activity of the CII domain. While we have yet to elucidate how CI-ATPase influences CII kinase activity, the results of this study identified a rather peculiar behaviour in the pressure dependence of KaiC-ATPase activity. When CI-ATPase activity and CII-ATPase activity were evaluated separately, the sum of the activity was less than the total activity of WT KaiC. Furthermore, this difference in activity was larger under higher pressure (Table 1). This residual activity might have been the result of mutual coupling between CI- and CII-ATPase through volume contraction, which appears to increase under high pressure.

In the present study, hydrostatic pressure was positively correlated with a reduction in the period length of the KaiC phosphorylation cycle. This phenomenon was the result of pressure-induced changes in ATPase activity and allosteric effects on the protein. It would be instructive to look at the phosphorylation and dephosphorylation rates to obtain a comprehensive understanding of how pressure affects the system. More generally, as expected, the temperature-invariant reaction varied with pressure because the proteins of organisms dwelling underground and in the upper water layers are not evolutionarily adapted to pressure-induced changes in molecular structure, dynamics, and stability. Subsequent pressure-axis experiments are expected to advance our current understanding of circadian rhythms, while, simultaneously, allowing us to elucidate the volumetric properties of the system.

## Materials and Methods

### Sample preparation

Recombinant Kai proteins (i.e. KaiA, KaiB, and KaiC) of the cyanobacterium *Synechococcus elongatus* PCC 7942 were synthesized using the conventional *E. coli* expression system, as reported previously^28^. The expression vector for the N-terminal domain fragment (CI-model, residues 2-247) of KaiC was generated from that of wild-type KaiC^29^. Expression vectors of KaiC-variants, E77Q/E78Q, R393C, and F470Y, were generated via the site-directed mutagenesis protocol^28^. Proteins were purified using a Strep-tactin Sepharose column (IBA GmbH, Göttingen, Germany), followed by liquid chromatography with SP Sepharose Fast Flow and superdex 75 PG 26/60 columns (GE Healthcare Co. Chicago, IL, USA). Protein concentration was determined via the Bradford method, using protein assay kits (FUJIFILM Wako Pure Co., Osaka, Japan) with bovine serum albumin (Bio-Rad Laboratories, Inc., Hercules, CA, USA) as the standard.

### High-pressure fluorescence spectroscopy

High-pressure fluorescence spectroscopic measurements were obtained using a spectrofluorometer (FP-8300; JASCO Co., Hachioji, Tokyo, Japan) and a pressure-resistant optical cell (Syn Co, Kyotonabe, Kyoto, Japan). KaiC (0.2 mg/mL) was co-incubated with KaiA (0.04 mg/mL) and KaiB (0.04 mg/mL) in 20 mM Tris-HCl buffer (pH 8.0) containing 150 mM NaCl, 6 mM ATP, and 5 mM MgCl_2_^4^. The protein solution was stored in a quartz inner cell. The excitation wavelength from a xenon arc lamp was set at 295 nm with a slit-width of 5 nm. Time-dependent tryptophan emission at 340 nm was measured at 900-s intervals from 1 bar to 200 bars at 30 °C. Temperature and pressure were maintained within ± 0.1 °C and ± 10 bars, respectively. The oscillation period was determined via a fast Fourier transformation (FFT) algorithm using the program IGOR Pro 6 (WaveMetrics Inc., Portland, OR, USA). All oscillations at a constant pressure could be primarily explained at a uniform frequency.

Tryptophan emission spectra of KaiA and KaiC were obtained from 1 bar to 300 bars at 30 °C and 310–450 nm, with a slit-width of 5 nm at a scanning speed of 200 nm/min. The excitation wavelength was set at 295 nm, with a slit-width of 5 nm. Tyrosine emission spectra of KaiB were obtained from 1 bar to 300 bars at 30 °C. Emission spectra at 290–380 nm were obtained with a slit-width of 5 nm and a scanning speed of 200 nm/min. The excitation wavelength was set at 280 nm, with a slit-width of 5 nm.

### Determination of KaiC ATPase activity

KaiC (0.2 mg/mL) in 20 mM Tris-HCl buffer (pH 8.0) containing 150 mM NaCl, 2 mM ATP, and 5 mM MgCl_2_ stored in silicon tubes was incubated in a pressure-resistant vessel (Syn Co, Kyotanabe, Kyoto, Japan) at varying pressures from 1 bar to 200 bars at 30 °C. Silicon tubes containing solution samples were removed from the vessel every 6 h and the ATPase activity of KaiC was measured using a high-performance liquid chromatography (HPLC) system with a Shim-Pack VP-ODS column (SHIMADZU GLC Ltd., Taito, Tokyo, Japan). The mobile phase used 100 mM phosphoric acid, 150 mM triethylamine, and 1% acetonitrile. ATP and ADP in reaction mixtures were separated at a flow rate of 0.4 mL/min. ATPase activity, *k*_cat_, was evaluated as a function of ADP production (i.e. linear slope/enzyme concentration). In Michaelis-Menten kinetics, assuming a pre-steady state reaction when [*S*] >> *K*_M_, where [*S*] and *K*_M_ are the substrate concentration and the Michaelis constant, respectively, the rate of product formation, *v*, is determined as follows:

1 $$v={k}_{cat}{[E]}_{0}$$

where [*E*]_0_ is the initial enzyme concentration. As reported previously^14^, the concentrations of enzyme and substrate used here satisfied the condition.

### Estimation of activation volumes

The rate constants under high pressure depend exponentially on the pressure and activation volumes for the reactions:

2 $$k(p)=k({p}^{0})\exp (-\frac{p{\rm{\Delta }}{V}^{\ne }}{RT})$$

where *p* and *p*^0^ represent high pressure and atmospheric pressure, respectively; ${\rm{\Delta }}{V}^{\ne }$, activation volume; *R*, gas constant; *T*, absolute temperature. Thus, the plots of ln *k*_cat_(*p*)/*k*_cat_(*p*^0^) versus *p* were fitted using linear least-squares analysis for the value of ${\rm{\Delta }}{V}^{\ne }$ (Fig. S4).

### Analysis of KaiC phosphorylation rhythm

KaiA (0.04 mg/mL), KaiB (0.04 mg/mL), and KaiC (0.2 mg/mL) in 20 mM Tris-HCl buffer (pH 8.0) containing 150 mM NaCl, 3 mM ATP, and 5 mM MgCl_2_ stored in silicon tubes was incubated at 1 bar and 200 bars (in the pressure-resistant vessel) at 30 °C. Silicon tubes containing solution samples were collected every 3 h. Temporal changes in KaiC phosphorylation ratios were analysed via SDS-PAGE and ImageJ (National Institutes of Health, Bethesda, MD, USA). We measured pressure-induced changes in the ratios of the phosphorylated KaiC to total KaiC in triplicate. In each experiment, SDS-PAGE was performed twice, and the mean of the KaiC phosphorylation ratios was calculated. The oscillation period was determined via the FFT algorithm using the program IGOR Pro 6 (WaveMetrics Inc., Portland, OR, USA). All oscillations at a constant pressure could be primarily explained at a uniform frequency.

## Supplementary information

Supplementary Information

Supplementary Dataset

## Data Availability

Figure source data are available from the journal web site.
