# Supplementary material for: Pressure accelerates the circadian clock of cyanobacteria
Source: Sci Rep. 2019 Aug 27;9:12395. doi: 10.1038/s41598-019-48693-1 (PMC6712028; doi:10.1038/s41598-019-48693-1)
Supplement: Supplementary file 1 — Supplementary Information [file 41598_2019_48693_MOESM1_ESM.pdf]

Supplementary Information

Title: Pressure accelerates the circadian clock of cyanobacteria

Authors: Ryo Kitahara[1,2,*], Katsuaki Oyama[2], Takahiro Kawamura[2], Keita Mitsuhashi[3], Soichiro Kitazawa[1], Kazuhiro Yasunaga[1], Natsuno Sagara[1], Megumi Fujimoto[2], and Kazuki Terauchi[2, 3]

Affiliations:
[1]College of Pharmaceutical Sciences, Ritsumeikan University, 1-1-1 Nojihigashi, Kusatsu, Shiga 525-8577, Japan.

[2]Graduate School of Life Sciences, Ritsumeikan University, 1-1-1 Nojihigashi, Kusatsu, Shiga 525-8577, Japan.

[3]College of Life Sciences, Ritsumeikan University, 1-1-1 Nojihigashi, Kusatsu, Shiga 525-8577, Japan.

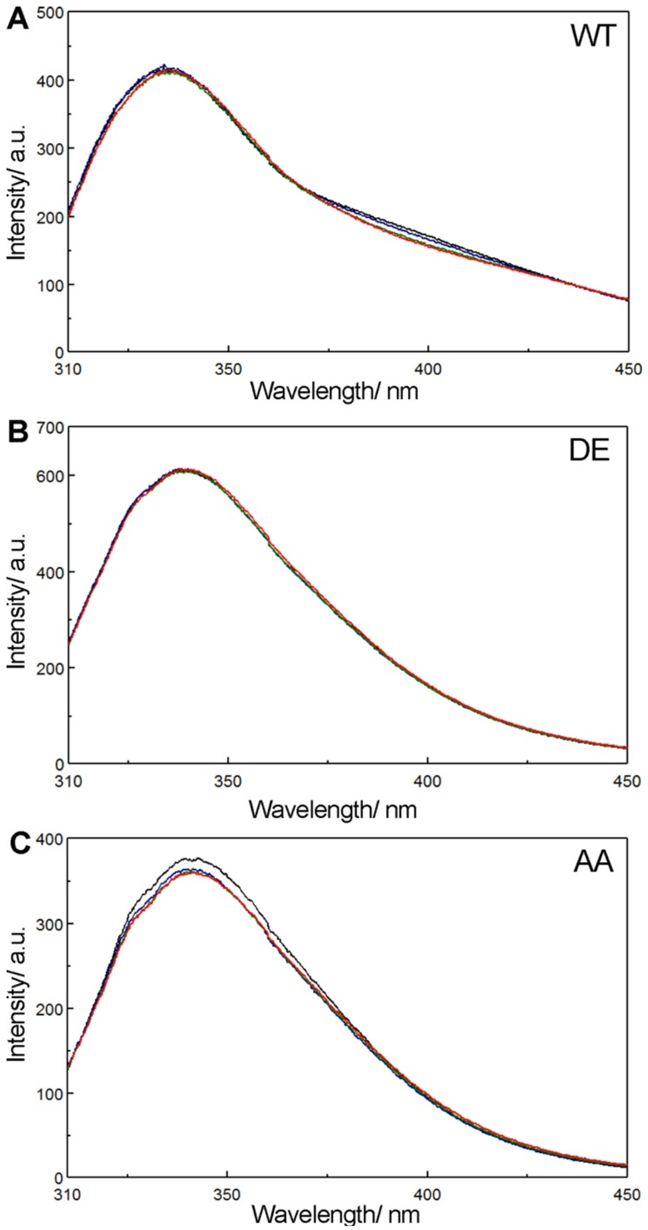

Fig. S1 Tryptophan fluorescence spectra of KaiC at different pressures at 30 °C. Spectra of KaiC-WT (A), -DE (S431D/T432E) (B), and -AA (S431A/T432A) (C) were obtained at 1 bar (black), 100 bars (blue), 200 bars (green), and 300 bars (red).

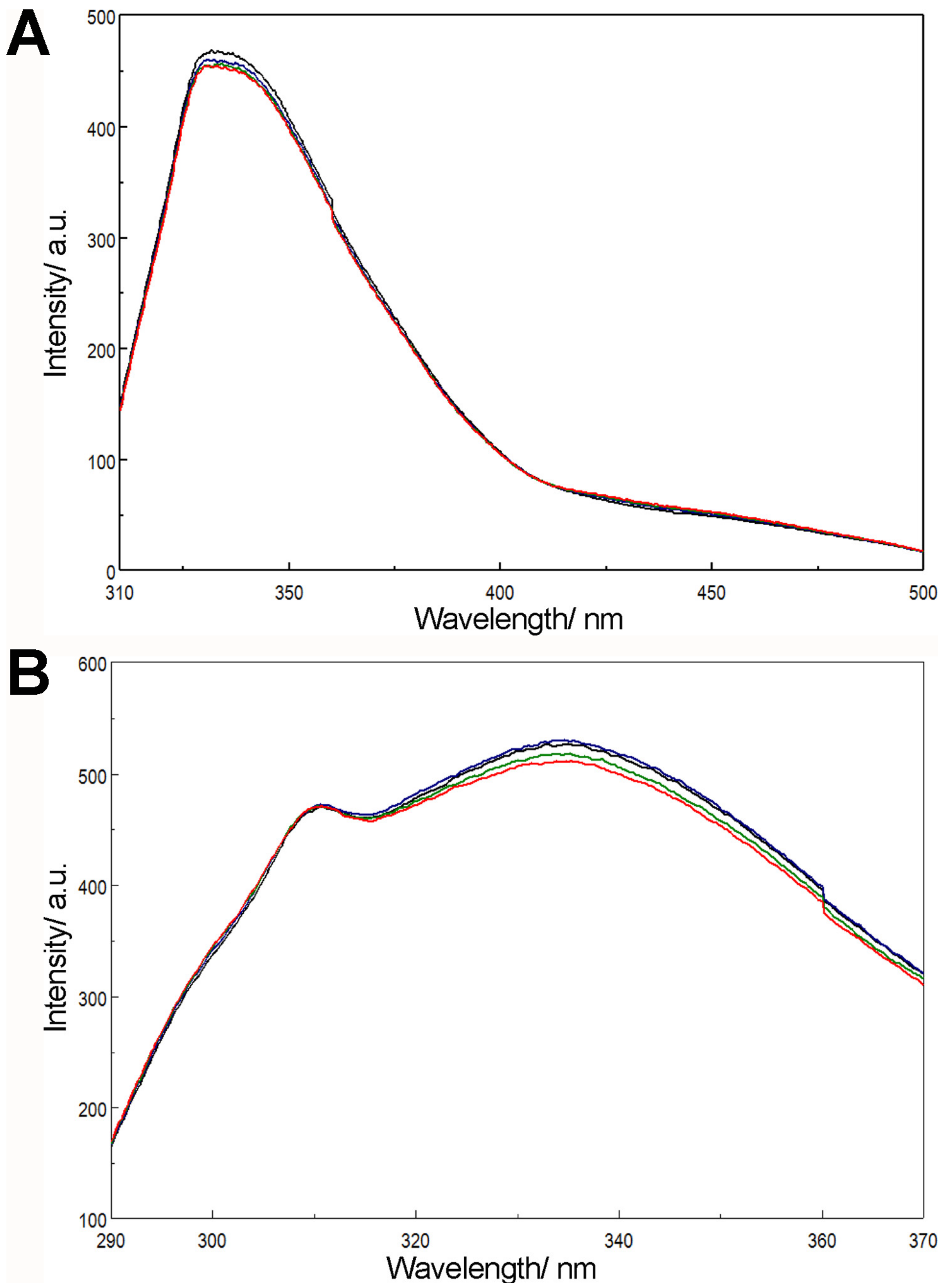

Fig. S2 Fluorescence emission of KaiA and KaiB at different pressures at 30 °C. (A) Tryptophan fluorescence spectra of KaiA at different pressures from 1 bar to 300 bars. (B) Tyrosine fluorescence spectra of KaiB at different pressures from 1 bar to 300 bars. Spectra were obtained at 1 bar (black), 100 bars (blue), 200 bars (green), and 300 bars (red).

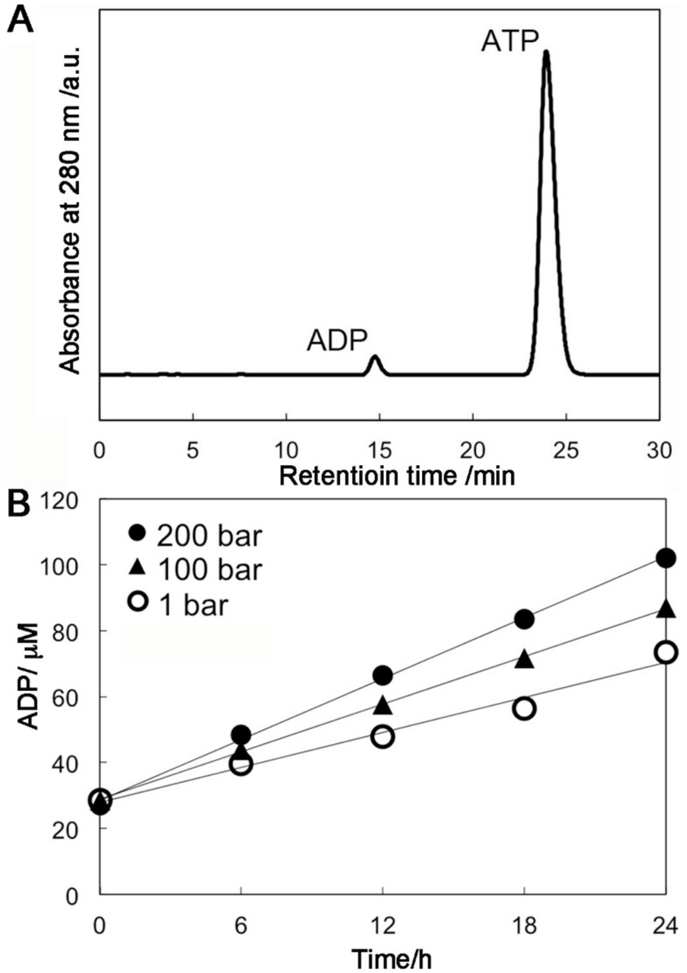

Fig. S3 Pressure dependence of KaiC ATPase activity. (A) HPLC analysis showing the separation of ADP and ATP. (B) Representative kinetics of ADP production by KaiC at 30 °C at 1 bar (open circles), 100 bars (closed triangles), and 200 bars (closed circles). Lines show the least-squares linear fit to data.

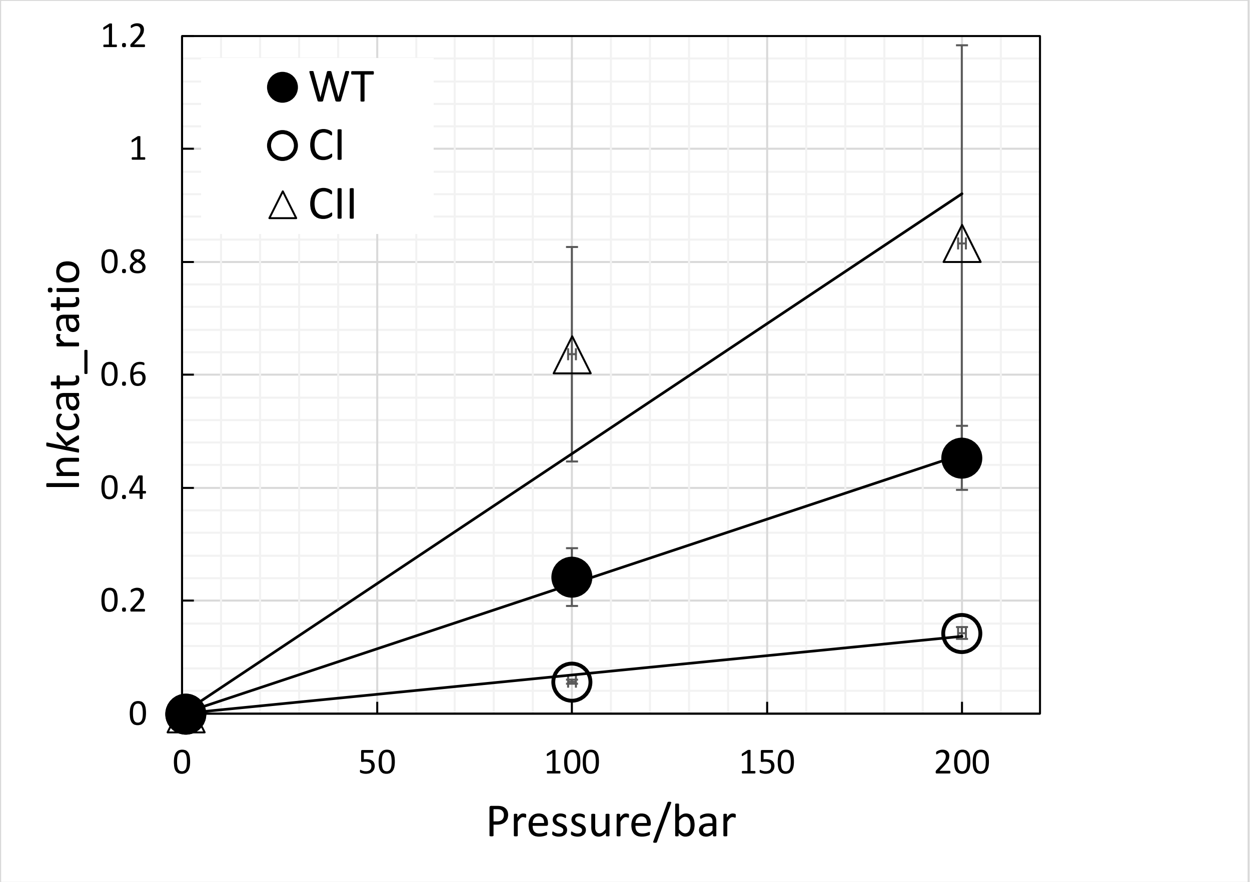

Fig. S4 Activation volumes of ATP hydrolysis by KaiC and its variants. WT (closed circles), CI (open circles), and CII (open triangles) show the wild-type KaiC, the N-terminal domain fragment (CI-model) of KaiC, and the E77Q/E78Q variant (CII-model) of KaiC, respectively. Lines show the least-squares linear fit.

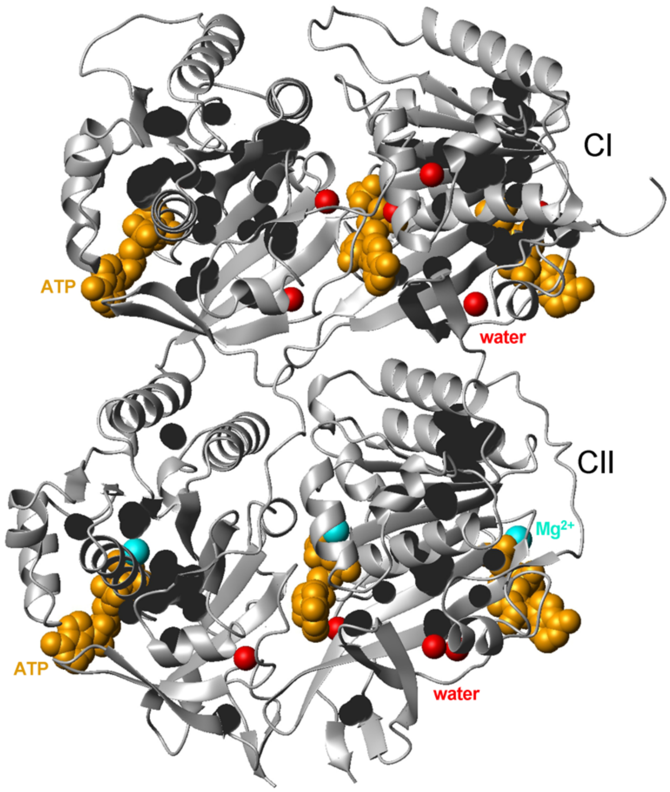

Fig. S5 Internal cavities of KaiC. The location of the cavity of the KaiC-ATP complex (PDB ID: 1U9I). Two subunits of KaiC (gray) and 6 ATP (gold) are depicted by ribbon and space-filling models, respectively. Mg^{+2} ions and water molecules are depicted by cyan and red spheres, respectively. Cavities, depicted by dark-blue spheres, were estimated using the MOLMOL program with a 1.4-Å probe size.
